# Risk score for esophageal and gastric cancer in the over 50-year-old population based on self-reported information –the RISC-GAP project

**DOI:** 10.1186/s12876-026-05069-w

**Published:** 2026-06-27

**Authors:** Timo Schmitz, Julia Reizner, Sha Sha, Ben Schöttker, Hermann Brenner, David Roser, Helmut Messmann, Christa Meisinger, Jakob Linseisen

**Affiliations:** 1 Epidemiology, Medical Faculty, University of Augsburg, Augsburg, Germany; 2 Division of Clinical Epidemiology and Early Cancer Detection, German Cancer Research Center, Heidelberg, Germany; 3 Department of Gastroenterology, University Hospital Augsburg, Augsburg, Germany

**Keywords:** Esophageal cancer, Gastric cancer, Screening, Risk score, Self-reported information

## Abstract

**Background:**

The aim was to build a risk score (RS) for gastric and esophageal cancer (GEC) based on self-reported information as a first step to develop a risk-adapted screening modality for GEC or precursor lesions in a non-high incidence region in the framework of the RISC-GAP project.

**Methods:**

Data from 375,280 participants aged 50 years and older in the UK Biobank project were used. The outcome was incident esophageal or gastric cancer. Various variables, including sociodemographic data, medical conditions, medication, lifestyle factors and diet, were initially considered. To be able to use the RS as a screening tool in the general population, only variables that can be determined by self-report were selected. For variable selection, we used COX regression models with LASSO penalization; the main criterion was 5- and 10-years AUC.

**Results:**

The final score included the following eight variables: sex, age, smoking status, drinking status, body mass index, history of esophagitis, medication with gastric acid inhibitors and surgery in the stomach/esophagus area. 10-fold cross-validation revealed a discrimination of 0.740 (5-year AUC) and 0.724 (10-year AUC), respectively. High-risk individuals were defined as those with a 10-year cancer risk of 1% or more (around 6% of the study population).

**Conclusion:**

The RS allows a reasonable discrimination of individuals with an elevated risk of gastric or esophageal cancer. In further steps of the RISC-GAP project it will be evaluated whether selection of a high-risk population can be further improved by additional clinical and biomarker information.

**Supplementary Information:**

The online version contains supplementary material available at 10.1186/s12876-026-05069-w.

## Background

Gastric and esophageal cancer (GEC) are major health threats to mainly elderly persons in Western societies [1, 2]. In 2020, gastric cancer incidence and mortality ranked 5th among total cancer incidence and mortality worldwide, and esophageal cancer incidence and mortality ranked 11th and 7th, respectively [3]. Over 70% of cases were diagnosed in Asian countries, and about 14% (gastric cancer) and 11% (esophageal cancer) in Europe [3]. In Germany, approximately 7,500 cases of esophageal cancer [4] and up to 15,000 cases of gastric cancer [5] are diagnosed every year. Annually, around 14,000 patients die due to either esophageal or gastric cancer in Germany [4, 5], which is mainly a consequence of a frequently late diagnosis, i.e., at advanced stages, and thus with a bad prognosis.

Although GEC incidence and mortality are far lower in Western countries than in many countries of Eastern Asia [1, 6] their early detection and treatment are of utmost importance, for the patients and the society. Unlike for breast, bowel, or prostate cancer, there exists no systematic preventive care program for these cancer entities in Germany. However, South Korea has already successfully established a nationwide prevention program for gastric cancer using endoscopic screening, which has led to a significant reduction in gastric cancer mortality of 41% [7–9]. Japan has implemented a similar prevention program, but its effectiveness is controversial [9]. In South Korea, every person over 40 years receives an invitation to a diagnostic gastroscopy every two years [8, 10]. However, gastroscopies are resource-intensive and carry a non-negligible risk of complications [11]. Since the lifetime risk of GEC is drastically lower in Germany than in South Korea, a general prevention program with an endoscopic examination for every person above a specific age range may be ineffective.

Rather, in middle- and low-incidence countries, a prevention program offered to individuals with a high risk seems to be a promising strategy. As a prerequisite, identification of a high-risk group for GEC is warranted. The goal of the project RISC-GAP (risk score-based screening of gastro-esophageal cancer and precursor lesions) is to develop a risk-adapted population-based screening program. This idea is also supported by recommendations of the European Commission which suggests to explore evidence-based strategies for gastric cancer prevention in European countries (EC-GaC) [12]. In the present study and as an initial step of the RISC-GAP project, data from the UK Biobank was used to calculate a risk score 1 (RS1) based on self-reported information provided by the UK biobank participants. The present work describes the steps taken to establish a RS1 based on variables that are most predictive of future GEC risk. The questions should be easy and quick to complete, and the score should allow a decent discrimination between persons with an elevated and a nont-elevated risk of GEC. In a subsequent screening step, individuals with a high risk according to the RS1 will then receive biomarker testing including predictive biomarker information to allow a more precise estimation of the GEC risk. One special marker of interest is Helicobacter pylori, as an infection with this bacterium is known to be a causal risk factor for gastric cancer [13].

## Materials and methods

### Study sample and sample size

The score building was based on data from the UK Biobank [14]. The dataset was selected from the database in October 2024. A total of 502,151 participants were examined at the baseline visit between 2006 and 2010. Each participant completed an extensive interview with questions on lifestyle factors (alcohol intake, smoking, physical activity, etc.), diet, preexisting diseases and comorbidities, and current medication. Additionally, every participant received a variety of physical examinations, e.g., bioimpedance measurements. A detailed description of the methods used for data collection at baseline can be found elsewhere [14, 15].

**Fig. 1:**
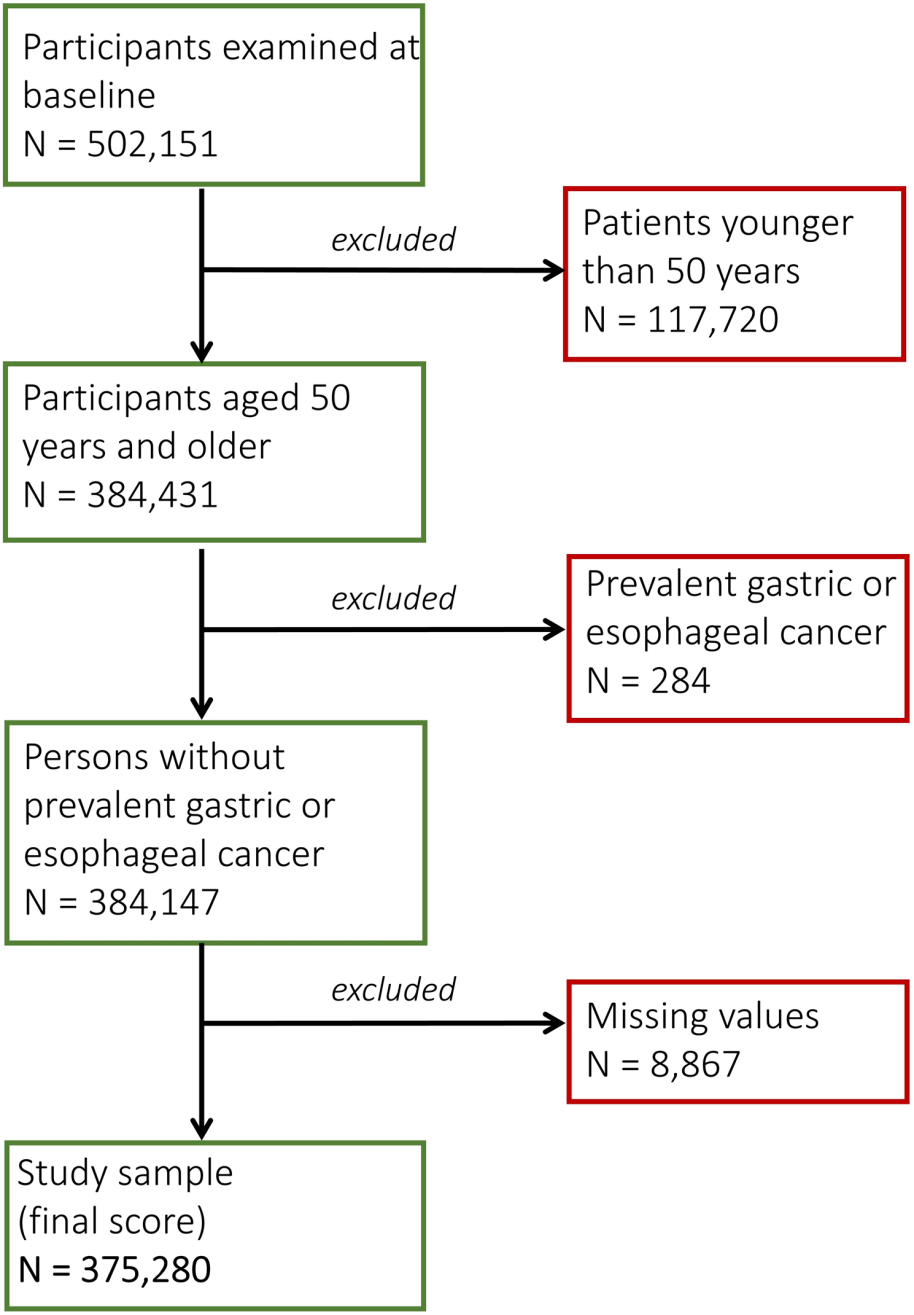
Flow chart displaying all inclusions/exclusions and the final sample size

For this specific analysis, we excluded all persons younger than 50 years and all persons with prevalent gastric or esophageal cancer at baseline (see Fig. 1). After these exclusions, 384,147 participants remained for the statistical analysis. Due to missing values in relevant variables, the final score was built on *N* = 375,280 participants.

### Outcome

The outcome of this analysis was gastric or esophageal cancer diagnosed after baseline visit. Therefore, the variables with the IDs 40,005 (date of cancer diagnosis) and 40,006 (ICD-10 of the cancer diagnosis), instances 1 to 21, were used. Gastric cancer was defined as ICD-10 C16, esophageal cancer was defined as ICD-10 C15. A combined event variable was created, which was set to 1 if a participant had a diagnosis of either gastric or esophageal cancer during follow-up and set to 0 if a participant had no such diagnosis until end of follow-up, loss to follow-up, or death.

Additionally, a time variable was created: if a participant had cancer during follow-up, the time between the baseline visit and the date of the diagnosis was assigned. Otherwise, if a patient died without a prior cancer diagnosis, the time variable was assigned the time between baseline visit and death. Information on death was extracted from the variables with the ID 40,000, instances 0 and 1. Otherwise, we used the censoring dates for cancer data, i.e. 31 December 2020 for England, 31 December 2016 for Wales, and 30 November 2021 for Scotland [16]. With this approach, we obtained a median follow-up time of 11.7 years [IQR: 10.9, 12.0]. As we were interested mainly in the risk of esophageal and gastric cancer occurring within the following 10 years, we censored the observational time to 12 years, which also avoids problems with violations of the proportional hazard assumption and issues with competing risk due to premature non-cancer death.

### Potential score variables

All variables considered for the score were based on information collected during baseline examination. Only such variables were considered that can be reported by most individuals of the population with an acceptable degree of validity. Table 1 displays all initially considered variables.

**Table 1:** Listing of all variables initially considered for the risk score

Variable	Coding/Categories
sociodemographics	
sex	male, female
age	years
ethnicity	white, non-white
Lifestyle	
alcohol drinking status	current drinker, ex-drinker, never drinker
smoking status	current smoker, ex-smoker, never smoker
BMI (body mass index)	kg/m² (the actual score askes for height and weight)
physical activity	high, moderate, low, no information
Medical conditions	
family history of colorectal cancer (parents, siblings)	yes, no
prior esophagitis	yes, no
prior gastritis	yes, no
asthma	yes, no
COPD (chronic obstructive pulmonary disease)	yes, no
stomach or abdominal pain*	yes, no
prior stomach or esophageal surgery	yes, no
Medication	
gastric acid inhibitor	regularly, not regularly
aspirin	regularly, not regularly
Diet	
vegetables (per day)	less than 3 tablespoons, 3–4 tablespoons, 5–6 tablespoons, more than 6 tablespoons
fresh fruits (per day)	less than 1 piece, 1–2 pieces, 3–6 pieces, more than 6 pieces
processed meat	never, less than once a week, once a week, twice a week or more
pork	never, less than once a week, once a week, twice a week or more
added salt to meals	never/rarely, sometimes, usually, always

* the exact question was: In the last month have you experienced stomach or abdominal pain that interfered with your usual activities?

### Statistical analysis

COX proportional hazard regression models with the least absolute shrinkage and selection operator (LASSO) penalization (L1 regularization) were used for variable selection and to minimize the risk of overfitting. First, we calculated LASSO models including all potential predictor variables (see Table 1). Two of the initial variables, age and BMI, were continuous variables. We tested for a non-linear relation using COX regression models and restricted cubic splines with 3 degrees of freedom. The association with age was regarded to be linear, while BMI showed a non-linear association. Therefore, a polynomial approach was used, including the original BMI variable as well as a squared BMI variable in the model.

On large data sets, apparent model performance is close to optimism-corrected performance [17, 18], so we chose to perform model construction on the whole data set. To assess actual model performance, we ran a 10-fold cross-validation with pre-specified variable selection, as the lambda for variable selection was not purely data-driven but also based on practical considerations. The main measure of model performance was 5- and 10-year AUC [19] and C index (Harrells C-Index or concordance index) [20].

Since the number of missing values in relevant variables was very low, we excluded all cases with missing information in any of the analyzed variables (complete case analysis). Due to the low number of missing values, we believe no relevant selection bias was introduced. Imputation of missing data was not performed.

The final model and its corresponding variables included was then displayed by a nomogram using the nomogram function from the R package rms [21]. It provides a visualization of the predictive values of each variable included into the final score model.

### Cut-off

All individuals with a 10-year GEC risk of 1% or higher according to the final score were classified as high-risk patients; these are supposed to receive further diagnostics in the scope of the RISC-GAP project. Kaplan-Meier estimator was used to calculate the 5- and 10-year absolute cancer risk for the total sample and the high-risk group.

### Additional analyses and score calculations

We calculated several other models in addition to the main model that was developed as described above. For all additional analysis, the variable selection was done a priori and COX models with LASSO penalization were used (for each model the lambda value associated with the best model performance was chosen).

First, we calculated simpler models using only the most predictive variables from the main model in order to evaluate model performance using fewer variables. The first model included only sex and age as predictors. The second model additionally included square-rooted BMI and smoking. For the latter model, we also calculated a model including these four variables and any possible two-variable interaction term to check for most relevant interactions.

Furthermore, we calculated the main model including also individuals younger than 50 years, to check if the score would potentially also fit for younger people.

Next, we built models separately for esophageal and gastric cancer to check whether investigating the different cancer locations separately would add any performance benefit.

Finally, we wanted to include information on family history (gastric or esophageal cancer in parents or siblings) in the score. Unfortunately, this information was not available in UK Biobank data. We decided to simulate such a variable using the following assumptions: the hazard ratio independent of all other variables in the score is 2.0 (doubled risk) [22, 23], and the prevalence of a positive family history is 4% (lifetime risk for gastric or esophageal cancer is about 2%) [4, 5]. Using these assumptions, we created a simulated variable (for further details, see supplementary Text 1) and included it in the score.

All analyses were performed using R version 4.4.1. The following essential packages were used: survival package, rms, glmnet, timeROC, DynNom, and survex.

## Results

### Baseline characteristics

Characteristics of the study sample are displayed in Table 2. Over the median follow-up time of 11.7 [IQR: 10.9–12.0] years, 1,145 cases of esophageal cancer and 767 cases of gastric cancer have been diagnosed. In the total sample, the mean age was 60.1 years, and a majority of 54.3% were women. Next to differences in other variables, patients who developed cancer during follow-up were significantly older, predominantly men, had a higher BMI, and were more likely to be smokers or ex-smokers.

**Table 2:** Baseline characteristics of the study sample. Patients younger than 50 years or with prevalent esophageal or gastric cancer were excluded

	Total sample (*N* = 384147)	No cancer group (*N* = 382245)	esophageal or gastric cancer during follow-up (*N* = 1902)	*P* value	N*
Follow-Up-Time (in years)	11.7 (10.9–12.0)	11.7 (10.9–12.0)	7.0 (3.8–9.6)	< 0.001	384,147
Esophageal cancer	1145 (0.3)	0 (0.0)	1145 (60.2)	< 0.001	384,147
Gastric cancer	767 (0.2)	0 (0.0)	767 (40.3)	< 0.001	384,147
Age at baseline (years, mean, SD)	60.1 (5.4)	60.1 (5.4)	62.3 (5.0)	< 0.001	384,147
Female	208,515 (54.3)	207,951 (54.4)	564 (29.7)	< 0.001	384,147
BMI (kg/m²)	26.9 (24.3–30.0)	26.9 (24.3–30.0)	28.0 (25.3–31.2)	< 0.001	381,807
Ethnicity				< 0.001	383,507
non-white	17,134 (4.5)	17,084 (4.5)	50 (2.6)		
white	366,373 (95.5)	364,524 (95.5)	1849 (97.4)		
Life-style factors					
Alcohol drinking status				< 0.001	382,980
never drinker	16,836 (4.4)	16,756 (4.4)	80 (4.2)		
ex-drinker	14,367 (3.8)	14,244 (3.7)	123 (6.5)		
current drinker	351,777 (91.9)	350,085 (91.9)	1692 (89.3)		
Smoking status				< 0.001	381,823
never smoker	201,504 (52.8)	200,840 (52.9)	664 (35.2)		
ex-smoker	143,846 (37.7)	142,948 (37.6)	898 (47.6)		
current smoker	36,473 (9.6)	36,147 (9.5)	326 (17.3)		
Physical activity				0.016	384,147
no information	94,165 (24.5)	93,688 (24.5)	477 (25.1)		
moderate	119,272 (31.0)	118,719 (31.1)	553 (29.1)		
high	117,100 (30.5)	116,537 (30.5)	563 (29.6)		
low	53,610 (14.0)	53,301 (13.9)	309 (16.2)		
Medical conditions					
Esophagitis	7184 (1.9)	7092 (1.9)	92 (4.8)	< 0.001	384,147
Gastritis	19,848 (5.2)	19,702 (5.2)	146 (7.7)	< 0.001	384,147
COPD	8864 (2.3)	8782 (2.3)	82 (4.3)	< 0.001	384,147
Asthma	44,073 (11.5)	43,831 (11.5)	242 (12.7)	0.093	384,147
Stomach or abdominal pain	29,778 (7.8)	29,603 (7.7)	175 (9.2)	0.020	384,147
Family history of colorectal cancer	45,558 (11.9)	45,320 (11.9)	238 (12.5)	0.396	384,147
Esophageal or gastric surgery	3876 (1.0)	3828 (1.0)	48 (2.5)	< 0.001	384,147
Medication					
Aspirin	63,204 (16.6)	62,763 (16.6)	441 (23.6)	< 0.001	379,756
Gastric acid inhibitors	33,132 (8.7)	32,871 (8.7)	261 (13.9)	< 0.001	379,756
Diet					
Vegetables (per day)				< 0.001	375,855
less than 3 tablespoons	60,561 (16.1)	60,178 (16.1)	383 (20.6)		
3–4 tablespoons	135,825 (36.1)	135,160 (36.1)	665 (35.8)		
5–6 tablespoons	101,728 (27.1)	101,260 (27.1)	468 (25.2)		
more than 6 tablespoons	77,741 (20.7)	77,401 (20.7)	340 (18.3)		
Fruits (per day)				< 0.001	381,885
less than 1 piece	31,936 (8.4)	31,706 (8.3)	230 (12.2)		
1–2 pieces	204,550 (53.6)	203,535 (53.6)	1015 (53.7)		
3–6 pieces	141,063 (36.9)	140,440 (37.0)	623 (33.0)		
more than 6 pieces	4336 (1.1)	4314 (1.1)	22 (1.2)		
Processed meat				< 0.001	382,603
never	34,855 (9.1)	34,714 (9.1)	141 (7.5)		
less than once a week	119,975 (31.4)	119,514 (31.4)	461 (24.4)		
once a week	112,512 (29.4)	111,947 (29.4)	565 (29.9)		
two times a week or more often	115,261 (30.1)	114,536 (30.1)	725 (38.3)		
Pork				< 0.001	380,989
never	61,102 (16.0)	60,825 (16.0)	277 (14.7)		
less than once a week	217,712 (57.1)	216,696 (57.2)	1016 (53.9)		
once a week	89,046 (23.4)	88,534 (23.4)	512 (27.1)		
two times a week or more often	13,129 (3.4)	13,048 (3.4)	81 (4.3)		
Salt added to meals				< 0.001	383,357
never/rarely	212,749 (55.5)	211,807 (55.5)	942 (49.6)		
sometimes	107,002 (27.9)	106,455 (27.9)	547 (28.8)		
usually	45,733 (11.9)	45,452 (11.9)	281 (14.8)		
always	17,873 (4.7)	17,744 (4.7)	129 (6.8)		

* Number of cases without missing values

### Score development

Initially, we calculated a model choosing the lambda (penalization) value associated with the best model performance (the lambda value corresponding to the minimum cross-validated error representing the optimal balance between model complexity and predictive performance), which resulted in a score with a total of 19 variables, see supplementary table S1. However, as we intended to build a more compact score with fewer variables, we stepwise increased the lambda. With increasing lambda values, ß-coefficients shrank and more and more variables were excluded. We continued to do so until we saw a significant drop in model performance (5- and 10-year area under the curve [AUC]), see supplementary Fig. 1.

**Fig. 2:**
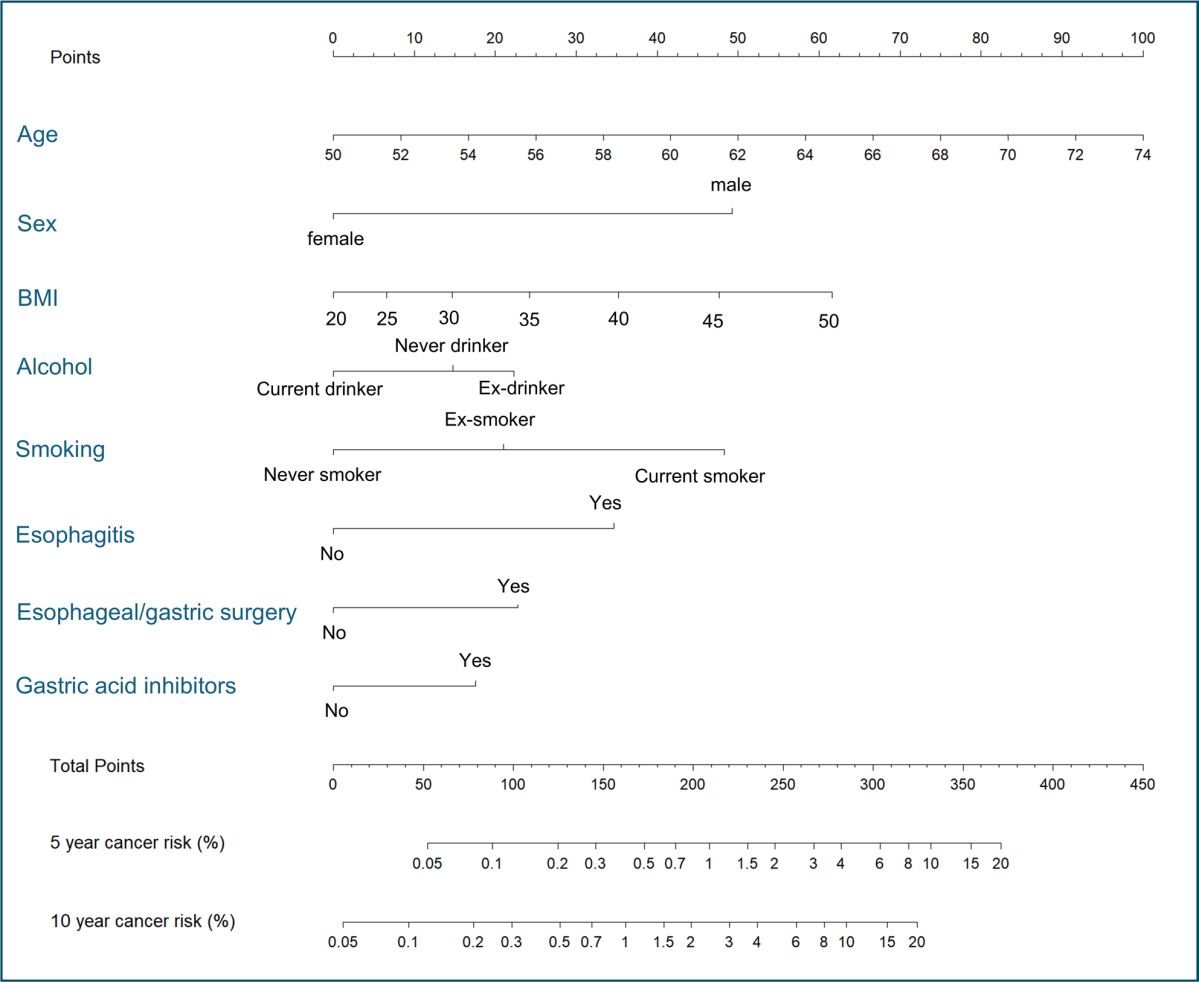
Nomogram of the final score displaying the predictive power of each variable. The numbers of each variable are added to a total sum. The cancer risk is then determined based on this sum

**Fig. 3:**
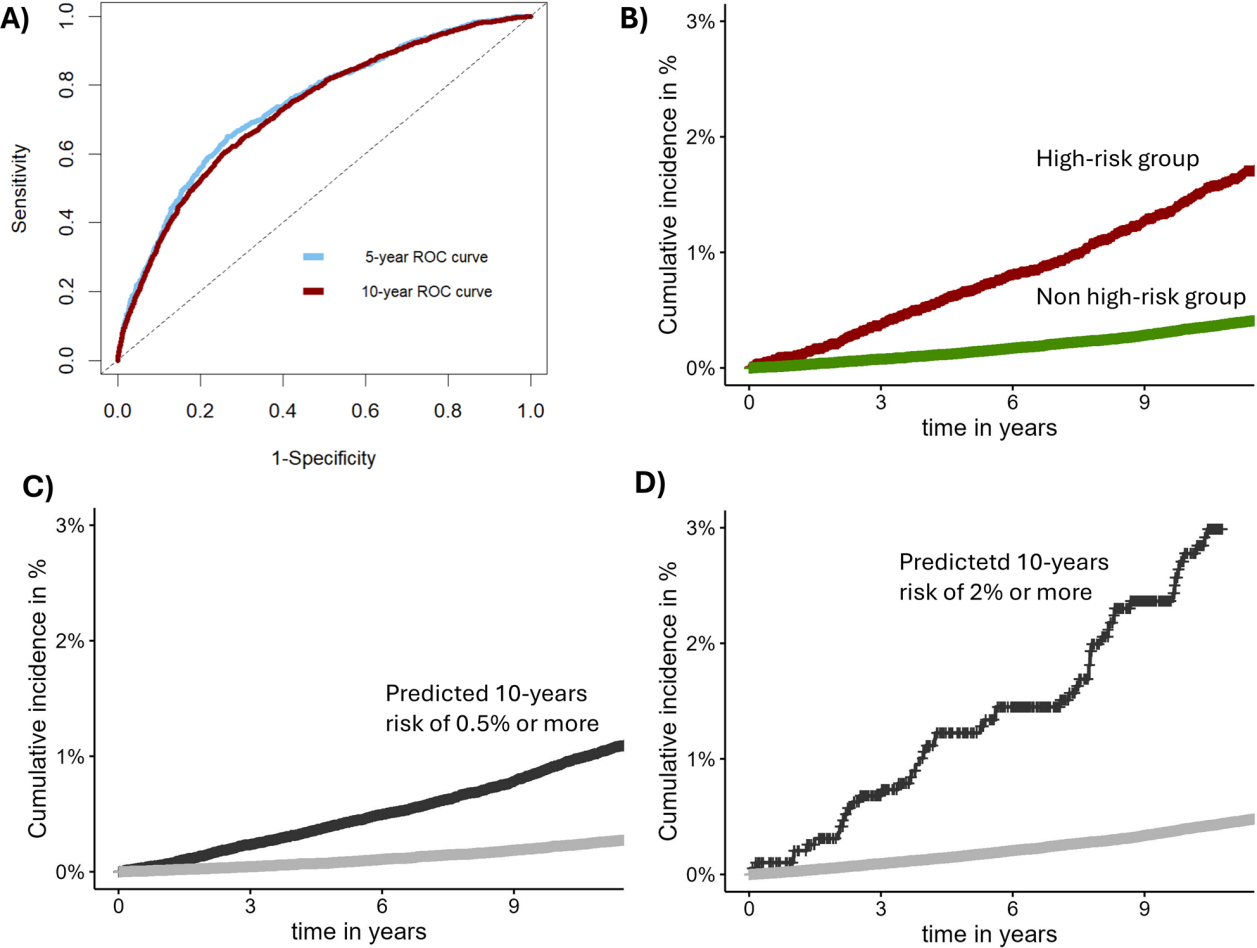
Discrimination of the final score. **A** 5- and 10-year AUC (apparent performance in the whole data set). **B** Kaplan-Meier curves for the high-risk patients (10-year cancer risk of 1% or more) and the non-high-risk patients. **C** and **D** Kaplan-Meier curves for alternative cut-offs (high risk group defined as all individuals with a predicted10-year cancer risk of 0.5% or more (**C)** and 2% or more (**D)**, respectively

As a very high lambda strongly reduced the ß-coefficients of the variables kept in the prediction score, we chose to calculate the final LASSO model only including the variables selected as described above and using the lambda associated with the best model performance, see Fig. 2 and supplementary table S1. The final model included the following variables: sex, age, alcohol drinking status, smoking status, BMI², history of esophagitis, surgery of the stomach/esophagus, and intake of gastric acid inhibitors (see Fig. 2). Table 3 displays the ß coefficients of each variable/category, which are summed up to calculate final score for each individual. The 10-fold-cross-validation model performance was 0.740 (5-year AUC) and 0.724 (10-year AUC), respectively (see Fig. 3A) and supplementary table S2). The Concordance-index (C-index) was 0.715. The cut-off value for the high-risk group (10-year cancer risk of 1% or more) was 7.0856 corresponding to 6.42% of the analyzed sample. Figure 3B) displays the cumulative incidence in the high-risk and the low-risk group. Figure 3C) and D) represent the curves for cumulative incidence when alternatively using 0.5% and 2% 10-year cancer risk as cut-off values for high-risk group definition. For the total sample, Kaplan-Meier estimator suggested a 5- year and 10-year cancer risk of 0.17% and 0.41% respectively. Numbers for the high-risk group were 0.64% and 1.46%, which corresponds to a 3.9-fold (5-year) and 3.6-fold (10-year) increase in cancer incidence in the high-risk group compared to the total group of individuals aged 50 years and older.

**Table 3:** Estimates (ß coefficients) of each included variable to calculate the final risk score

Variable	ß coefficient (LASSO)	Corresponding Hazard ratio
Age (year)	0.078154684	1.08
Sex (male)	0.925959156	2.52
BMI² (kg/m²)	0.000581035	1.00
Alcohol – never drinker	0.216461979	1.24
Alcohol – former drinker	0.462499038	1.59
Smoking – ex-smoker	0.383312906	1.47
Smoking – smoker	0.929689435	2.53
Esophagitis	0.665204379	1.94
Esophageal or gastric surgery	0.504457626	1.66
Gastric acid inhibitors	0.309694966	1.36

Figure S2 (supplementary material) displays the calibration of the model within the training data frame (calibration line 5 year: y = -0.031 + 1.208x; 10 year: y = -0.031 + 1.098x). The data shows that the predicted risk and the actual risk are close to one another for every 10%-quantile of the risk score (linear predictor term). Supplementary table S3 displays the characteristics of the included score variables stratified by high and low risk classification according to the final score.

### Additional analyses

The model including only sex and age already reaches a 10-fold-cross-validation performance of 0.711 (5-year AUC) and 0.697 (10-year AUC), which demonstrates that sex and age are the two main predictors of the score, see supplementary table S4 and S5 and supplementary figure S3 (A). Additionally, including BMI and smoking further improves prediction performance, which then is already close to the performance of the main score (supplementary table S4 and S5 and figure S3 (B)). Including all possible two-variable interaction terms into the model with sex, age, BMI and smoking does not substantially improve prediction (supplementary table S4), so we concluded there are no major interactions between the most important score predictors which could have improved prediction performance.

The model performance of the main score model on the data set also including patients younger than 50 years improves the performance relevantly (5-year AUC: 0.775, 10-year AUC: 0.759, see supplementary table S4 and S5), which is consequence of a more heterogeneous study sample but also proves that the score would be suitable for younger patients as well.

When calculating the model performances with the predefined variables from the main score separately for the outcomes gastric cancer and esophageal cancer, the 10-fold cross-validation performances are close to those seen for the combined outcome (esophageal cancer: 5-year AUC: 0.748, 10-year: 0.740, c-index: 0.727; gastric cancer: 5-year AUC: 0.732, 10-year: 0.710, c-index: 0.703). Supplementary Table S6 shows the models for the separated outcomes including all initially considered variables (Table 1). No relevant improvement in model performance or selected covariables was observed, which confirmed us to model the main score using a combined outcome.

Finally, the performance and beta coefficients of the main model supplemented by the simulated family history variable (supplementary text 1) are presented in supplementary table S7 and S8.

## Discussion

In this study, we intended to develop a useful and well-discriminating risk score for esophageal and gastric cancer based on self-reported information. The score building was based on data from the UK Biobank, and - as the target group for the RISC-GAP project [24] was 50 years and older - we included only persons aged 50 years or older. The risk score was required to be compact and handy, so we limited the number of predictors to 10. The final score included the variables age, sex, BMI^2^, smoking status, alcohol drinking status, history of esophagitis, medication with gastric acid inhibitors and prior surgery of the esophagus or stomach. The score reached a discrimination value of 0.740 (5-year AUC) and 0.724 (10-year AUC).

### Comparison to the previously published risk scores

Over the last two decades, other risk scores for gastric [25] and/or esophageal cancer [26] have been developed. However, there is great heterogeneity across the scores regarding to study sample and sample size, country, used methodology, purpose of use, and so on, which limits the overall comparability. The discriminatory power of the scores varies quite heavily, with a range for the C-statistics from 0.71 to 0.88 for esophageal cancer [26]. According to a systematic review of gastric cancer risk scores, the AUC ranged from 0.68 to 0.93 [25]. Compared to these data, the discrimination of the present score is in the lower mid-range. One main reason for this is the use of only self-reported information from study participants. To our knowledge, there is only a very limited number of scores that were based only on self-reported information. The second main reason might be the inclusion of individuals aged 50 years and above. Including also younger persons with a considerably lower cancer risk leads to a more heterogeneous study sample and increases the discriminatory power considerably. Furthermore, the very large sample size in the present study should be emphasized, which reduces the risk of overfitting. Consequently, the large sample size decreases the performance of the score in the development data set but increases performance in cohorts other than the UK Biobank.

A further difference in the present score compared to most previously published scores is the combined endpoint of gastric and/or esophageal cancer. Most scores concentrate on either one or even on a specific histological subtype (adenocarcinoma/squamous cell carcinoma) or a specific location (cardia, pylorus, etc.). Here, both cancer types were used as a combined outcome, as both share common risk factors and, in both cases, upper gastrointestinal endoscopy is the gold-standard diagnostic procedure for intended screening. We developed risk score models for both cancer types separately and found great agreement in both models, so we decided to model the score with a combined outcome.

### Selected predictors

The two most important predictors in the present study were sex and age, with older men having by far the highest risk. Older age and male sex are well-known risk factors for esophageal [2, 27] and gastric cancer [28] and the majority of existing risk scores have identified them as major predictors as well [25, 26, 29].

Smoking is one of the most important avoidable risk factors for gastric [30, 31] and esophageal cancer [32]. This is also represented by our score, as smoking was found to be one of the most predictive variables apart from sex and age.

The same can be said about overweight and obesity, which also increased the risk of the two specific cancer entities examined in this study [31, 33–35]. However, the association found was non-linear but appropriately modeled with a quadratic term (BMI^2^). This represents the circumstance that especially very obese individuals have a considerably increased risk of developing gastric or esophageal cancer.

Like smoking, alcohol consumption is considered a major preventable risk factor for a variety of cancer types in the digestive tract, including cancer in the mouth [36], esophageal cancer (squamous cell carcinoma) [37], gastric cancer [30, 38] and colorectal cancer [39]. In the present study, the alcohol drinking status (current, previous, never) was part of the final score. However, the current drinker group had the lowest cancer risk which somehow contradicts the concept of alcohol being a risk factor. An important aspect to consider in this regard is that we only tested the frequency of alcohol intake but did not test the actual amount (in grams) consumed per day or week. This information was skipped as collecting this data would have greatly complicated the questionnaire. Prior literature results indicated that the association between alcohol consumption and the risk of esophageal [2, 40] and gastric [41] cancer might be strongly dose-dependent. In a study by Pandeya et al. it was reported that persons who drank moderate levels of wine, port, or spirits had a lower risk of esophageal cancer compared to nondrinkers [40], which fits with our results. Furthermore, it can be speculated whether the group of ex-drinkers contains a considerable proportion of individuals who stopped drinking alcohol according to health complaints, which would potentially explain their elevated risk. It remains unclear, however, why persons who never drank alcohol occasionally or regularly have an increased cancer risk, too. Miss-reporting by persons who consumed rather high amounts of alcohol but did not want the declare this could be one explanation. Also, the association between never drinking alcohol and increased cancer risk might represent unknown confounding rather than a causal association (alcohol as a proxy variable). For example Asian people have a less effective alcohol metabolism which likely affects their alcohol consumption [42], but also have an increased gastric and esophageal cancer risk [43]. Another circumstance to consider is that never drinkers might predominantly share a specific cultural or ethnic background (alcohol consumption in the Arabic world for instance is potentially lower than in European countries [44]). So, the increased cancer risk might be caused rather by ethnicity or group specific lifestyle patterns but not by alcohol abstinence itself.

Two important variables that were selected for the final score are the history of esophagitis and medication with gastric acid inhibitors. While smoking, alcohol, or obesity can be described as general risk factors with detrimental effects on various aspects of health, prior esophagitis and medication with gastric acid inhibitors can be considered more specific predictors of gastric or esophageal cancer. Esophagitis was also part of two previously published scores for esophageal cancer [45, 46]. To minimize miss-reporting, the corresponding question was phrased properly and symptom-oriented.

As existing literature suggests, proton pump inhibitors (PPI), especially when used longer than 3 months, might be associated with an increased risk of gastric and esophageal cancer [47]. There is less evidence for H2R blocker [47]. However, the variable ‘medication with gastric acid inhibitors’ (including PPI and H2R blockers) being part of the final risk score is supported by existing literature.

The last variable that was selected by the LASSO model was surgery in the stomach or esophagus area. Gastric or esophageal surgery had also been mentioned in existing literature as a potential risk factor for gastric cancer [48]. To the best of our knowledge, neither patients who have a history of esophagitis, esophageal or gastric surgery or who take gastric acid inhibitors are automatically under any form of regular surveillance in the UK or German health system. However, it appears plausible that these patients are under closer observation regarding diseases in the upper GI which might affect the likelihood of detecting esophageal or gastric cancer.

Finally, current literature suggests a positive family history as a major risk factor for GEC. Prior studies indicated a duplication in risk for persons with a positive family history [22, 23]. Thus, ‘family history of esophageal or gastric cancer’ (parents and siblings only) was meant to be included in the risk score. However, this specific information was not available in the UK Biobank data. The combined lifetime risk for gastric or esophageal cancer in Germany is approximately 2.9% for men and 1.4% for women [4, 5], so we assumed that about 4% of all individuals over 50 years should have a positive family history. With these two assumptions (hazard ratio of 2 [independent of other risk factors] and 4% of all individuals with positive family history), we simulated such a variable accordingly. This variable was then included as a risk predictor in an additional score, which is presented in the supplementary material.

### Non-selected predictors

A multitude of previous studies identified a variety of other risk factors and predictors of gastric and/or esophageal cancer [25, 26]. As discussed above, some of these suggested risk factors had made it into the final model of the score. However, others were not as predictive as expected from previous literature. Among these risk factors and potential predictors is dietary salt intake, which showed a positive association with gastric cancer in a recent meta-analysis (pooled odds ratio of 1.55) [49]. Moreover, other dietary habits and foods like red meat or vegetables have been reported to be associated with either esophageal [37] or gastric cancer [50] but yet, did not make it into the final risk score. The same can be said about ethnicity [51–53] which is supposed to affect the risk of gastric or esophageal cancer, however, was not part of the final score. Finally, aspirin intake was reported to be associated with lower gastric an esophageal cancer risk yet did not make it into the final model [54, 55]. Even though many of these variables represent important risk or protective factors and might actually be associated causally with cancer risk, their predictive value (independent of other important predictors) was only moderate and therefore the variables were not selected for the final models. It can be supposed that parts of the effects of these variables are already conveyed by the variables that are part of the final score.

### LASSO regression

For score building, we decided to use COX proportional hazard regression with LASSO penalization for variable selection and to avoid overfitting [56, 57]. The LASSO penalization is a relatively new method and is often used to build prediction and risk scores. As for any regression method, one receives ß coefficients and the score for any individual can be calculated by multiplying these coefficients with the person’s specific characteristics. This can be easily implemented when building a specific application that individuals can use to determine their gastric and esophageal cancer risk.

### Setting the cut-off for defining a high-risk group

The risk score developed in the present study is intended to be used as a first step in a risk-adapted screening procedure for esophageal and gastric cancer in over 50-years-old persons in Germany. A person identified as high risk person is then, in a second screening step, supposed to receive comprehensive biomarker testing that includes predictive biomarkers. Furthermore, information taken from medical charts may be included to obtain a more precise assessment of the specific cancer risk. As in each high-risk approach, persons with a non-high risk are not offered the screening examinations. Thus, exclusion from the high-risk group in the first step, i.e., using risk score 1, does not allow the persons to enter the next level, i.e., the estimation of a summary risk score including further information. With these considerations in mind, we decided to define a person as a high-risk person if the 10-year cancer risk was 1.0% or above, which corresponds to the upper 6.42% of all UK Biobank participants over 50 years with the highest 10-year cancer risk. Kaplan-Meier curves confirmed a clear separation in GEC risk when using this cut-off level (Fig. 3B).

### 5- and 10-year risk of gastric and esophageal cancer

In Germany, the risk of gastric cancer is higher compared to the risk of esophageal cancer [4]. Astonishingly, the situation is exactly the opposite in the UK [58], which is also confirmed by the observed numbers of cases in the UK Biobank. The reason for this circumstance remains mainly unclear. However, the incidence for both cancer sites is somewhere around four times lower in Western Europa compared to Western Asia [43]. In the latter, a general endoscopy-based cancer screening is proven effective [7, 8]. In the high-risk group defined by our score, the incidence of esophageal and gastric cancer is around four times higher compared to the total sample and should therefore be in the same range as in the general population in Western Asia. This suggests that the identified high-risk sample is indeed suitable as a starting point for an endoscopy-based cancer screening.

### Strengths and limitations

This score is characterized by some major strengths. First, it is based on a very large number of well-characterized persons participating in the UB biobank. Second, the median follow-up time of 11.7 years is longer than for most other studies. The score is based purely on information that almost all persons in the general population can provide with a high degree of validity. The included variables with their specific characteristics are kept very simple, and the calculation of the score can be implemented easily, which makes the score suitable for broad use.

However, there are some limitations to consider. Based on a comprehensive literature search, various variables were tested as risk predictors. It cannot be excluded though that important variables were missed, e.g., because information was not captured by the UK Biobank. We used LASSO regression for variable selection, but other methods might have chosen different variables. Although we have checked for interactions between the most important predictors (age, sex, BMI, smoking), we could have also missed some relevant interaction between the predictors (e.g., between smoking and alcohol). The score was built on individuals living in the UK (single-country study); direct transferability to other countries might not be possible without further ado. Finally, the score was not validated on an external data set.

## Conclusions

In the present study, we developed a risk score for esophageal and gastric cancer in the over-50-year-old population in a low-to-middle incidence area. The score includes eight variables that can be self-reported by most individuals. The score shows decent discrimination and is intended to identify high-risk persons defined by the 10-year risk of 1% or higher. Whether this risk score is suitable for selecting a high-risk screening population or needs additional clinical and biomarker information for better prediction and discrimination of a high-risk group will be addressed in further steps of the RISC-GAP project. The proof of the ability of the screening procedure to decrease GEC mortality at acceptable costs will decide about its implementation.

## Supplementary Information

Supplementary Material 1.

## Data Availability

The dataset supporting the conclusions of this article is available in the UK Biobank repository, https://ukbiobank.dnanexus.com.

## Abbreviations

AUC: Area under the curve
C index: Concordance index
GEC: Gastric and esophageal cancer
LASSO: Least absolute shrinkage and selection operator
PPI: Proton pump inhibitors
RS: Risk score
RS1: Risk score 1

## References

[1] López MJ, Carbajal J, Alfaro AL, Saravia LG, Zanabria D, Araujo JM, et al. Characteristics of gastric cancer around the world. Crit Rev Oncol/Hematol. 2023;181:103841. 10.1016/j.critrevonc.2022.103841. 36240980 10.1016/j.critrevonc.2022.103841

[2] Uhlenhopp DJ, Then EO, Sunkara T, Gaduputi V. Epidemiology of esophageal cancer: update in global trends, etiology and risk factors. Clin J Gastroenterol. 2020;13:1010–21. 10.1007/s12328-020-01237-x. 32965635 10.1007/s12328-020-01237-x

[3] International Agency for Research on Cancer (WHO). Global Cancer Observatory: IARC. 2024. https://gco.iarc.fr/en.

[4] Robert Koch-Institut, Herausgeber und die Gesellschaft der epidemiologischen Krebsregister in Deutschland e.V, Herausgeber. Krebs in Deutschland für 2019/2020 - Ösophaguskrebs. 2023. https://www.krebsdaten.de/Krebs/DE/Content/Publikationen/Krebs_in_Deutschland/kid_2023/kid_2023_c15_speiseroehre.pdf?__blob=publicationFile.

[5] Robert Koch-Institut, Herausgeber und die Gesellschaft der epidemiologischen Krebsregister in Deutschland e.V, Herausgeber. Krebs in Deutschland für 2019/2020 - Magenkrebs. 2023. https://www.krebsdaten.de/Krebs/DE/Content/Publikationen/Krebs_in_Deutschland/kid_2023/kid_2023_c16_magen.pdf?__blob=publicationFile.

[6] Morgan E, Soerjomataram I, Rumgay H, Coleman HG, Thrift AP, Vignat J, et al. The Global Landscape of Esophageal Squamous Cell Carcinoma and Esophageal Adenocarcinoma Incidence and Mortality in 2020 and Projections to 2040: New Estimates From GLOBOCAN 2020. Gastroenterology. 2022;163:649–e6582. 10.1053/j.gastro.2022.05.054. 35671803 10.1053/j.gastro.2022.05.054

[7] Kim B, Cho S-J. Endoscopic Screening and Surveillance for Gastric Cancer. Gastrointest Endosc Clin N Am. 2021;31:489–501. 10.1016/j.giec.2021.03.004. 34053635 10.1016/j.giec.2021.03.004

[8] Suh Y-S, Lee J, Woo H, Shin D, Kong S-H, Lee H-J, et al. National cancer screening program for gastric cancer in Korea: Nationwide treatment benefit and cost. Cancer. 2020;126:1929–39. 10.1002/cncr.32753. 32031687 10.1002/cncr.32753

[9] Sun D, Mülder DT, Li Y, Nieboer D, Park JY, Suh M, et al. The Effect of Nationwide Organized Cancer Screening Programs on Gastric Cancer Mortality: A Synthetic Control Study. Gastroenterology. 2024;166:503–14. 10.1053/j.gastro.2023.11.286. 38007053 10.1053/j.gastro.2023.11.286

[10] Kim T-H, Kim I-H, Kang SJ, Choi M, Kim B-H, Eom BW, et al. Korean Practice Guidelines for Gastric Cancer 2022: An Evidence-based, Multidisciplinary Approach. J Gastric Cancer. 2023;23:3–106. 10.5230/jgc.2023.23.e11. 36750993 10.5230/jgc.2023.23.e11 PMC9911619

[11] Levy I, Gralnek IM. Complications of diagnostic colonoscopy, upper endoscopy, and enteroscopy. Best practice & research. Clin Gastroenterol. 2016;30:705–18. 10.1016/j.bpg.2016.09.005. 10.1016/j.bpg.2016.09.005 27931631

[12] European Commission. Initiative on Gastric Cancer (EC-GaC). https://cancer-screening-and-care.jrc.ec.europa.eu/en/ec-gac. Accessed 2026.

[13] Reyes VE. Helicobacter pylori and Its Role in Gastric Cancer. Microorganisms. 2023. 10.3390/microorganisms11051312. 10.3390/microorganisms11051312 PMC10220541 37317287

[14] Sudlow C, Gallacher J, Allen N, Beral V, Burton P, Danesh J, et al. UK biobank: an open access resource for identifying the causes of a wide range of complex diseases of middle and old age. PLoS Med. 2015;12:e1001779. 10.1371/journal.pmed.1001779. 25826379 10.1371/journal.pmed.1001779 PMC4380465

[15] UK Biobank Coordinating Centre. UK Biobank: Protocol for a large-scale prospective epidemiological resource. 2007. https://www.ukbiobank.ac.uk/media/gnkeyh2q/study-rationale.pdf.

[16] UK Biobank. Cancer censoring dates. https://biobank.ndph.ox.ac.uk/ukb/exinfo.cgi?src=Data_providers_and_dates. Accessed January 2025.

[17] Steyerberg EW. Validation in prediction research: the waste by data splitting. J Clin Epidemiol. 2018;103:131–3. 10.1016/j.jclinepi.2018.07.010. 30063954 10.1016/j.jclinepi.2018.07.010

[18] Steyerberg EW. Clinical Prediction Models: A Practical Approach to Development, Validation, and Updating. 2nd ed. Cham: Springer International Publishing AG; 2019.

[19] Heagerty PJ, Lumley T, Pepe MS. Time-dependent ROC curves for censored survival data and a diagnostic marker. Biometrics. 2000;56:337–44. 10.1111/j.0006-341x.2000.00337.x. 10877287 10.1111/j.0006-341x.2000.00337.x

[20] Pencina MJ, D’Agostino RB. Overall C as a measure of discrimination in survival analysis: model specific population value and confidence interval estimation. Stat Med. 2004;23:2109–23. 10.1002/sim.1802. 15211606 10.1002/sim.1802

[21] Frank Harrell. nomogram: Draw a Nomogram Representing a Regression Fit. https://www.rdocumentation.org/packages/rms/versions/6.8-2/topics/nomogram. Accessed 2026.

[22] Peters Y, van Grinsven E, Siersema PD. Systematic review with meta-analysis: the effects of family history on the risk of Barrett’s oesophagus and oesophageal adenocarcinoma. Aliment Pharmacol Ther. 2021;54:868–79. 10.1111/apt.16558. 34383966 10.1111/apt.16558 PMC9292032

[23] Song M, Camargo MC, Weinstein SJ, Best AF, Männistö S, Albanes D, Rabkin CS. Family history of cancer in first-degree relatives and risk of gastric cancer and its precursors in a Western population. Gastric Cancer. 2018;21:729–37. 10.1007/s10120-018-0807-0. 29455268 10.1007/s10120-018-0807-0 PMC7380686

[24] Epidemiologie Augsburg. RISC-GAP. 2025. https://www.uni-augsburg.de/de/fakultaet/med/profs/epidemiologie/RISC-GAP/.

[25] Gu J, Chen R, Wang S-M, Li M, Fan Z, Li X, et al. Prediction Models for Gastric Cancer Risk in the General Population: A Systematic Review. Cancer Prev Res (Phila). 2022;15:309–18. 10.1158/1940-6207.CAPR-21-0426. 35017181 10.1158/1940-6207.CAPR-21-0426

[26] Li H, Sun D, Cao M, He S, Zheng Y, Yu X, et al. Risk prediction models for esophageal cancer: A systematic review and critical appraisal. Cancer Med. 2021;10:7265–76. 10.1002/cam4.4226. 34414682 10.1002/cam4.4226 PMC8525074

[27] Liu C-Q, Ma Y-L, Qin Q, Wang P-H, Luo Y, Xu P-F, Cui Y. Epidemiology of esophageal cancer in 2020 and projections to 2030 and 2040. Thorac Cancer. 2023;14:3–11. 10.1111/1759-7714.14745. 36482832 10.1111/1759-7714.14745 PMC9807450

[28] Yang W-J, Zhao H-P, Yu Y, Wang J-H, Guo L, Liu J-Y, et al. Updates on global epidemiology, risk and prognostic factors of gastric cancer. World J Gastroenterol. 2023;29:2452–68. 10.3748/wjg.v29.i16.2452. 37179585 10.3748/wjg.v29.i16.2452 PMC10167900

[29] Wong MCS, Leung EY-M, Yau STY, Chan SC, Xie S, Xu W, Huang J. Prediction algorithm for gastric cancer in a general population: A validation study. Cancer Med. 2023;12:20544–53. 10.1002/cam4.6629. 37855240 10.1002/cam4.6629 PMC10660462

[30] Machlowska J, Baj J, Sitarz M, Maciejewski R, Sitarz R. Gastric Cancer: Epidemiology, Risk Factors, Classification, Genomic Characteristics and Treatment Strategies. Int J Mol Sci. 2020. 10.3390/ijms21114012. 32512697 10.3390/ijms21114012 PMC7312039

[31] Karimi P, Islami F, Anandasabapathy S, Freedman ND, Kamangar F. Gastric cancer: descriptive epidemiology, risk factors, screening, and prevention. Cancer Epidemiol Biomarkers Prev. 2014;23:700–13. 10.1158/1055-9965.EPI-13-1057. 24618998 10.1158/1055-9965.EPI-13-1057 PMC4019373

[32] Wang Q-L, Xie S-H, Li W-T, Lagergren J. Smoking Cessation and Risk of Esophageal Cancer by Histological Type: Systematic Review and Meta-analysis. J Natl Cancer Inst. 2017. 10.1093/jnci/djx115. 29933436 10.1093/jnci/djx115

[33] Li Q, Zhang J, Zhou Y, Qiao L. Obesity and gastric cancer. Front Biosci (Landmark Ed). 2012;17:2383–90. 10.2741/4059. 22652786 10.2741/4059

[34] Schlottmann F, Dreifuss NH, Patti MG. Obesity and esophageal cancer: GERD, Barrett´s esophagus, and molecular carcinogenic pathways. Expert Rev Gastroenterol Hepatol. 2020;14:425–33. 10.1080/17474124.2020.1764348. 32441160 10.1080/17474124.2020.1764348

[35] Cho JH, Shin CM, Han K-D, Yoon H, Park YS, Kim N, Lee DH. Abdominal obesity increases risk for esophageal cancer: a nationwide population-based cohort study of South Korea. J Gastroenterol. 2020;55:307–16. 10.1007/s00535-019-01648-9. 31792601 10.1007/s00535-019-01648-9

[36] Ogden GR. Alcohol and oral cancer. Alcohol. 2005;35:169–73. 10.1016/j.alcohol.2005.04.002. 16054978 10.1016/j.alcohol.2005.04.002

[37] Qin X, Jia G, Zhou X, Yang Z. Diet and Esophageal Cancer Risk: An Umbrella Review of Systematic Reviews and Meta-Analyses of Observational Studies. Adv Nutr. 2022;13:2207–16. 10.1093/advances/nmac087. 36041184 10.1093/advances/nmac087 PMC9776643

[38] Ma K, Baloch Z, He T-T, Xia X. Alcohol Consumption and Gastric Cancer Risk: A Meta-Analysis. Med Sci Monit. 2017;23:238–46. 10.12659/msm.899423. 28087989 10.12659/MSM.899423 PMC5256369

[39] Zhou X, Wang L, Xiao J, Sun J, Yu L, Zhang H, et al. Alcohol consumption, DNA methylation and colorectal cancer risk: Results from pooled cohort studies and Mendelian randomization analysis. Int J Cancer. 2022;151:83–94. 10.1002/ijc.33945. 35102554 10.1002/ijc.33945 PMC9487984

[40] Pandeya N, Williams G, Green AC, Webb PM, Whiteman DC. Alcohol consumption and the risks of adenocarcinoma and squamous cell carcinoma of the esophagus. Gastroenterology. 2009;136:1215–24. e1-2. 19250648 10.1053/j.gastro.2008.12.052

[41] Tramacere I, Negri E, Pelucchi C, Bagnardi V, Rota M, Scotti L, et al. A meta-analysis on alcohol drinking and gastric cancer risk. Ann Oncol. 2012;23:28–36. 10.1093/annonc/mdr135. 21536659 10.1093/annonc/mdr135

[42] Wall TL, Ehlers CL. Genetic Influences Affecting Alcohol Use Among Asians. Alcohol Health Res World. 1995;19:184–9. 31798054 PMC6875758

[43] Bray F, Ferlay J, Soerjomataram I, Siegel RL, Torre LA, Jemal A. Global cancer statistics 2018: GLOBOCAN estimates of incidence and mortality worldwide for 36 cancers in 185 countries. CA Cancer J Clin. 2018;68:394–424. 10.3322/caac.21492. 30207593 10.3322/caac.21492

[44] AlMarri TSK, Oei TPS. Alcohol and substance use in the Arabian Gulf region: a review. Int J Psychol. 2009;44:222–33. 10.1080/00207590801888752. 22029498 10.1080/00207590801888752

[45] Chen W, Li H, Ren J, Zheng R, Shi J, Li J, et al. Selection of high-risk individuals for esophageal cancer screening: A prediction model of esophageal squamous cell carcinoma based on a multicenter screening cohort in rural China. Int J Cancer. 2021;148:329–39. 10.1002/ijc.33208. 32663318 10.1002/ijc.33208

[46] Xie S-H, Lagergren J. A model for predicting individuals’ absolute risk of esophageal adenocarcinoma: Moving toward tailored screening and prevention. Int J Cancer. 2016;138:2813–9. 10.1002/ijc.29988. 26756848 10.1002/ijc.29988

[47] Sawaid IO, Samson AO. Proton Pump Inhibitors and Cancer Risk: A Comprehensive Review of Epidemiological and Mechanistic Evidence. J Clin Med. 2024. 10.3390/jcm13071970. 38610738 10.3390/jcm13071970 PMC11012754

[48] Joshi SS, Badgwell BD. Current treatment and recent progress in gastric cancer. CA Cancer J Clin. 2021;71:264–79. 10.3322/caac.21657. 33592120 10.3322/caac.21657 PMC9927927

[49] Wu X, Chen L, Cheng J, Qian J, Fang Z, Wu J. Effect of Dietary Salt Intake on Risk of Gastric Cancer: A Systematic Review and Meta-Analysis of Case-Control Studies. Nutrients. 2022. 10.3390/nu14204260. 10.3390/nu14204260 PMC9609108 36296944

[50] Maddineni G, Xie JJ, Brahmbhatt B, Mutha P. Diet and carcinogenesis of gastric cancer. Curr Opin Gastroenterol. 2022;38:588–91. 10.1097/MOG.0000000000000875. 36165035 10.1097/MOG.0000000000000875

[51] Gupta S, Tao L, Murphy JD, Camargo MC, Oren E, Valasek MA, et al. Race/Ethnicity-, Socioeconomic Status-, and Anatomic Subsite-Specific Risks for Gastric Cancer. Gastroenterology. 2019;156:59–e624. 10.1053/j.gastro.2018.09.045. 30267713 10.1053/j.gastro.2018.09.045 PMC6309455

[52] Brown LM. The role of race/ethnicity in the epidemiology of esophageal cancer. J Assoc Acad Minor Phys. 2000;11:32–7. 10953542

[53] Corona E, Yang L, Esrailian E, Ghassemi KA, Conklin JL, May FP. Trends in Esophageal Cancer Mortality and Stage at Diagnosis by Race and Ethnicity in the United States. Cancer Causes Control. 2021;32:883–94. 10.1007/s10552-021-01443-z. 34003396 10.1007/s10552-021-01443-z PMC8236464

[54] Niikura R, Hirata Y, Hayakawa Y, Kawahara T, Yamada A, Koike K. Effect of aspirin use on gastric cancer incidence and survival: A systematic review and meta-analysis. JGH Open. 2020;4:117–25. 10.1002/jgh3.12226. 32280753 10.1002/jgh3.12226 PMC7144786

[55] Song Y, Zhong X, Gao P, Zhou C, Shi J, Wu Z, et al. Aspirin and Its Potential Preventive Role in Cancer: An Umbrella Review. Front Endocrinol (Lausanne). 2020;11:3. 10.3389/fendo.2020.00003. 32038497 10.3389/fendo.2020.00003 PMC6989406

[56] TIBSHIRANI R, THE LASSO METHOD FOR VARIABLE SELECTION IN, THE COX MODEL. Stat Med. 1997;16:385–95. 10.1002/(sici)1097-0258(19970228)16:4&lt;385::aid-sim380&gt;3.0.co;2-3. 9044528 10.1002/(sici)1097-0258(19970228)16:4<385::aid-sim380>3.0.co;2-3

[57] TIBSHIRANI R. Regression Shrinkage and Selection Via the Lasso. J Royal Stat Soc Ser B: Stat Methodol. 1996;58:267–88. 10.1111/j.2517-6161.1996.tb02080.x.

[58] Cancer research UK. Cancer statistic for the UK. 2017–2019. https://www.cancerresearchuk.org/health-professional/cancer-statistics-for-the-uk.

