# Supplementary material for: Risk score for esophageal and gastric cancer in the over 50-year-old population based on self-reported information –the RISC-GAP project
Source: BMC Gastroenterol. 2026 Jun 27;26:387. doi: 10.1186/s12876-026-05069-w (PMC13312655; doi:10.1186/s12876-026-05069-w)
Supplement: Supplementary file 1 — Supplementary Material 1. [file 12876_2026_5069_MOESM1_ESM.pdf]

Risk score for esophageal and gastric cancer in the over 50-year-old population based on self-reported information –the RISC-GAP project.

Timo Schmitz, Julia Reizner, Sha Sha, Ben Schöttker, Hermann Brenner, David Roser, Helmut Messmann, Christa Meisinger, Jakob Linseisen

Table S1: Results of the initial LASSO models (all potential predictors included). On the left the model using the optimum penalty, on the right using a higher lambda value (see Figure S1).

	Optimum penalty model (λ = 0.00005993)	***Model with higher lambda (λ=0.0005530844)***
Apparent Performance		
5-year AUC:	0.7467957	0.7380558
10-year-AUC:	0.7328971	0.7252033
Concordance-Index:	0.7232511	0.715375
10-fold-Cross-Validation Performance		
5-year AUC:	0.7419961	0.7358773
10-year-AUC:	0.7296878	0.7238843
Concordance-Index:	0.7198333	0.7140208
Optimism		
5-year AUC:	0.0047996	0.0021785
10-year-AUC:	0.0032093	0.001319
Concordance-Index:	0.0034178	0.0013542
Selected Variables	sex, age, ethnicity, alcohol, smoking status, BMI^2, physical activity, esophagitis, gastritis, asthma, COPD, stomach/abdominal pain, stomach/esophageal surgery, gastric acid inhibitors, vegetables, fresh fruit, processed meat, pork, added salt	sex, age, alcohol, smoking status, BMI^2, esophagitis, stomach/esophageal surgery, gastric acid inhibitors

Table S2: Model performance of the final LASSO models including only variables selected by the initial LASSO model (optimum penalty models). The model included the following predictors: sex, age, alcohol, smoking status, BMI^2, esophagitis, stomach/esophageal surgery, gastric acid inhibitors.

	Main Model (λ = 0.00004127048)
Apparent Performance	
5-year AUC:	0.7410382
10-year-AUC:	0.7258414
Concordance-Index:	0.7166834
10-fold-Cross-Validation Performance	
5-year AUC:	0.7397784
10-year-AUC:	0.724353
Concordance-Index:	0.7153994
Optimism	
5-year AUC:	0.0012598
10-year-AUC:	0.0014884
Concordance-Index:	0.001284

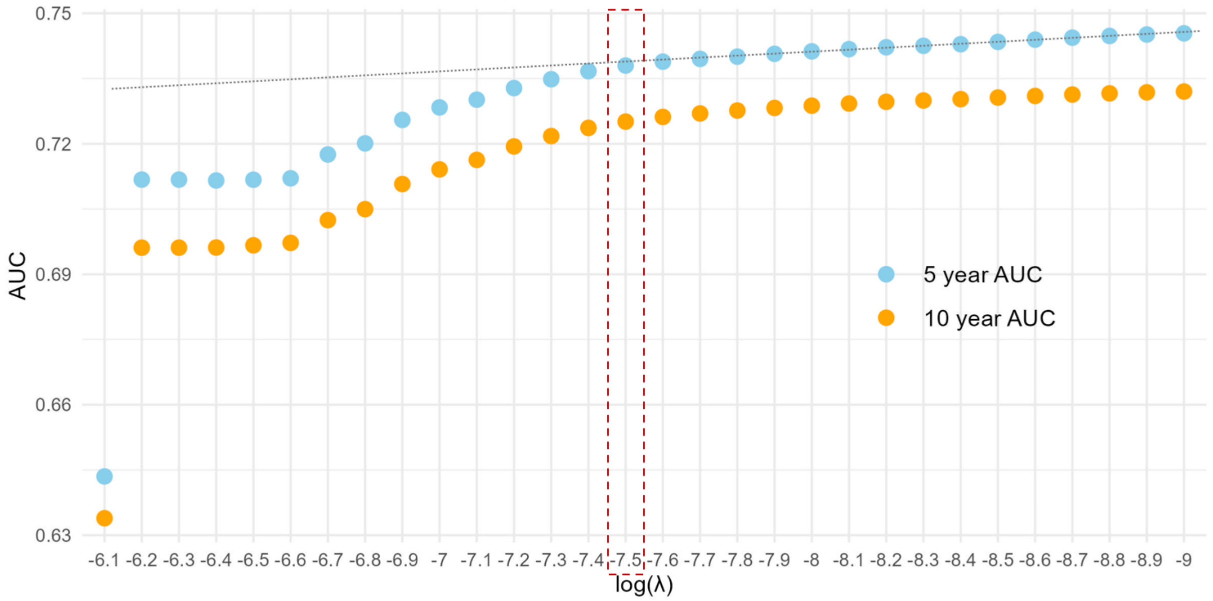

Figure S1: AUC values for different λ in the LASSO models. We chose a log(λ) of -7.5 for variable selection, since at this value the curve has an inflection point with a more than linear decrease of AUC with increasing λ.

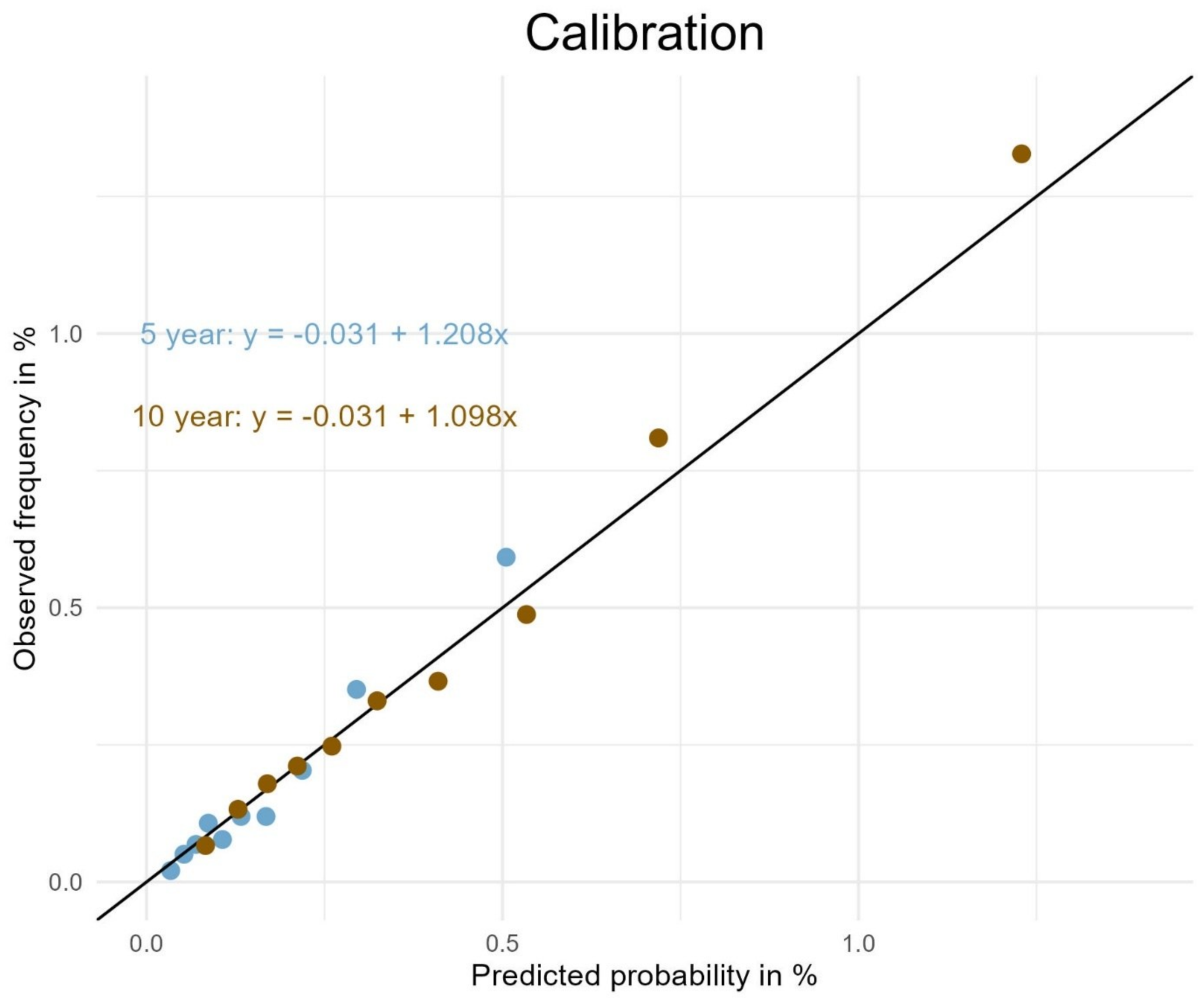

Figure S2: Predicted vs. observed cancer risk. The data frame was stratified in 10% quantiles according to the linear predictor. The blue dots represent 5-year-cancer risk and the brown dots represent 10-year-cancer risk. The figure demonstrates that the model is well calibrated for the UK-Biobank data frame.

Supplementary table S3: Characteristics of the score variables stratified by persons classified as high and low risk respectively.

	Total sample (N=375280)	***Risk score***		***P value***
		high risk (N= 24085)	***non-high risk (N= 351195)***	
Score	5.8 (5.3 - 6.5)	7.3 (7.2 - 7.5)	5.8 (5.3 - 6.3)	<0.001
Age at baseline (mean, SD)	60.1 (5.4)	65.5 (3.4)	59.7 (5.4)	<0.001
Male	171105 (45.6)	23339 (96.9)	147766 (42.1)	<0.001
BMI (kg/m²)	26.9 (24.3 - 30.0)	29.5 (26.7 - 32.9)	26.7 (24.2 - 29.8)	<0.001
Smoking status				<0.001
never smoker	198181 (52.8)	1746 (7.2)	196435 (55.9)	
ex smoker	141589 (37.7)	13261 (55.1)	128328 (36.5)	
current smoker	35510 (9.5)	9078 (37.7)	26432 (7.5)	
Alcohol drinking status				<0.001
never drinker	16151 (4.3)	812 (3.4)	15339 (4.4)	
ex-drinker	13876 (3.7)	3017 (12.5)	10859 (3.1)	
current drinker	345253 (92.0)	20256 (84.1)	324997 (92.5)	
Esophagitis	6928 (1.8)	2882 (12.0)	4046 (1.2)	<0.001
Esophageal or gastric surgery	3753 (1.0)	1301 (5.4)	2452 (0.7)	<0.001
Gastric acid inhibitors	32671 (8.7)	6334 (26.3)	26337 (7.5)	<0.001

Supplementary table S4: Different models and the corresponding model performances

	Sex and age model	***Model including sex, age, smoking, and BMI***	***Model including sex, age, smoking, BMI, and all two-variable interaction terms***	***Main model including persons younger than 50 years***
Apparent Performance				
5-year AUC:	0.711153619	0.734109285	0.736581716	0.775634085
10-year-AUC:	0.696847908	0.721120348	0.72354426	0.760106922
Concordance-Index:	0.687687615	0.710898653	0.713670527	0.749247805
10-fold-Cross-Validation Performance				
5-year AUC:	0.711149486	0.734719059	0.734627543	0.774514139
10-year-AUC:	0.696711955	0.720769734	0.721521073	0.758899336
Concordance-Index:	0.687375524	0.71073353	0.711544683	0.748021477
Optimism				
5-year AUC:	0.000004133	-0.000609774	0.001954173	0.001119946
10-year-AUC:	0.000135953	0.000350614	0.002023187	0.001207586
Concordance-Index:	0.000312091	0.000165123	0.002125844	0.001226328

Supplementary Table S5: Estimates (ß coefficients) of different models

Variable	***ß coefficient (LASSO)***		
	Sex and age score	***Score including sex, age, smoking, and BMI***	***Main model, including persons younger than 50 years***
Age (year)	0.077337125	0.080223175	0.083422397
Sex (male)	0.999665499	0.916065109	0.913259557
BMI^2 (kg/m^2)	-	0.00063972	0.000585708
Smoking – ex-smoker	-	0.393177549	0.37477388
Smoking – smoker	-	0.937549704	0.906944053
Alcohol – never drinkers		-	0.20021091
Alcohol – former drinker		-	0.445355289
Esophagitis	-	-	0.719456411
Esophageal or gastric surgery	-	-	0.489840457
Gastric acid inhibitors	-	-	0.301711368

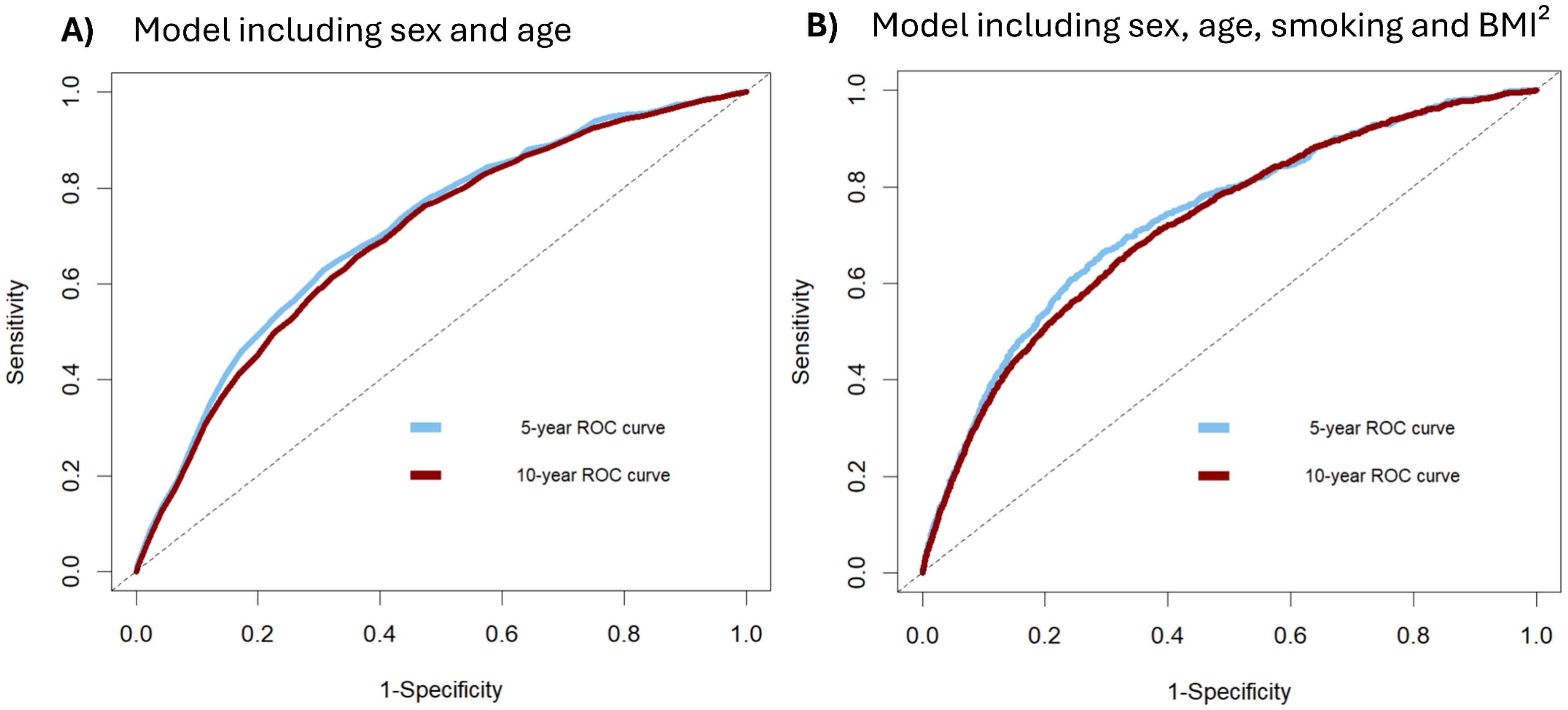

Supplementary figure S3: ROC curves for the models including only sex and age (A) and the model additionally including BMI^2 and smoking (B).

Supplementary Table S6: Models with separate outcomes (esophageal and gastric cancer). Considered variables are those presented in table 1.

	Esophageal cancer	***Gastric cancer***
Apparent Performance		
5-year AUC:	0.758768309	0.738559072
10-year-AUC:	0.751334059	0.717903063
Concordance-Index:	0.739016972	0.713255415
10-fold-Cross-Validation Performance		
5-year AUC:	0.750502508	0.732910085
10-year-AUC:	0.742980297	0.710935286
Concordance-Index:	0.731095336	0.705920223
Optimism		
5-year AUC:	0.008265801	0.005648987
10-year-AUC:	0.008353762	0.006967777
Concordance-Index:	0.007921636	0.007335192
Variables selected by LASSO regression		
	sex, age, ethnicity, alcohol, smoking status, BMI^2, physical activity, esophagitis, asthma, COPD, stomach/esophageal surgery, family history of colorectal cancer, gastric acid inhibitors, ASS, vegetables, fresh fruit, processed meat, pork, added salt	sex, age, alcohol, smoking status, BMI^2, gastritis, stomach or abdominal pain, stomach/esophageal surgery, family history of colorectal cancer, ASS, vegetables, fresh fruit, processed meat, added salt

Supplementary Text 1: Simulation of the variable ‚Family history of gastric or esophageal cancer‘

Assumptions:

1) Life-time risk of gastric or esophageal cancer about 2%
   → about 4% of patients have a positive family history
2) Persons with a positive family history have a doubled risk of cancer independent of other (selected) covariables

4-field board

	Family history	No Family history	
Cancer	A = 142	B = 1,708	A+B = 1,850
No cancer	C =14,869	D = 358,561	C+D = 373,430
	A+C = 0.04·375,280	B+D = 0.96·375,280	N = 375,280

Assumption: Persons with a family history of esophageal or gastric cancer have a doubled risk of cancer themselves, independent of other covariables selected for the model. This assumption translates into the following equation:

$$\frac{A}{A+C} = 2\frac{B}{B+D} \qquad \Rightarrow \qquad A = \frac{2\,(A+B)(A+C)}{(B+D)+2(A+C)}$$

Simulated Variable

Using the 4-field-bord above, we build a variable in the following manner:

- In the group of persons with cancer (N= 1,850), 142 persons were randomly assigned with a positive family history. The remaining 1,708 cancer patients were assigned with no family history
- Likewise, in the group of individuals without cancer during follow-up (N=373,430), 14,869 persons were randomly assigned with a positive family history and the remaining 358,561 were assigned to the no family history group.

Supplementary Table S7: Estimates (ß coefficients) of each variable of the risk score, including the simulated family history variable.

Variable	***ß coefficient (LASSO)***
Age (year)	0.078244838
Sex (male)	0.926309593
BMI^2 (kg/m^2)	0.000583815
Alcohol – never drinker	0.221234584
Alcohol – former drinker	0.462129582
Smoking – ex-smoker	0.385217444
Smoking – smoker	0.934119473
Esophagitis	0.659911455
Gastric acid inhibitors	0.314163591
Esophageal or gastric surgery	0.506852391
Family history (first degree) of esophageal or gastric cancer	0.6641912352

Table S8: Model performance of the LASSO models, including the predictors of the main model and additionally the simulated family history variable.

	Model including simulated family history
Apparent Performance	
5-year AUC:	0.7432645
10-year-AUC:	0.7315848
Concordance-Index:	0.7220438
10-fold-Cross-Validation Performance	
5-year AUC:	0.7414036
10-year-AUC:	0.7298845
Concordance-Index:	0.72002
Optimism	
5-year AUC:	0.0018609
10-year-AUC:	0.0017003
Concordance-Index:	0.0020238
